# Levonorgestrel releasing intrauterine system (Mirena) versus endometrial ablation (Novasure) in women with heavy menstrual bleeding: a multicentre randomised controlled trial

**DOI:** 10.1186/1472-6874-13-32

**Published:** 2013-08-08

**Authors:** Malou C Herman, Marian J van den Brink, Peggy M Geomini, Hannah S van Meurs, Judith A Huirne, Heleen P Eising, Anne Timmermans, Johanna MA Pijnenborg, Ellen R Klinkert, Sjors F Coppus, Theodoor E Nieboer, Ruby Catshoek, Lucet F van der Voet, Hugo WF van Eijndhoven, Giuseppe CM Graziosi, Sebastiaan Veersema, Paul J van Kesteren, Josje Langenveld, Nicol AC Smeets, Huib AAM van Vliet, Jan Willem van der Steeg, Yvonne Lisman-van Leeuwen, Janny H Dekker, Ben W Mol, Marjolein Y Berger, Marlies Y Bongers

**Affiliations:** 1 Department of Obstetrics & Gynaecology, Máxima Medical Centre, PO Box 777, 5500, Veldhoven, MB, The Netherlands; 2 Department of General Practice, University of Groningen, UMCG, Groningen, The Netherlands; 3 Department of Obstetrics & Gynaecology, AMC, Amsterdam, The Netherlands; 4 Department of Obstetrics & Gynaecology, VU Medical Centre, Amsterdam, The Netherlands; 5 Department of Obstetrics & Gynaecology, Gelre Ziekenhuis, Apeldoorn, The Netherlands; 6 Department of Obstetrics & Gynaecology, Twee Steden Ziekenhuis, Tilburg, The Netherlands; 7 Department of Obstetrics & Gynaecology, UMCG, Groningen, The Netherlands; 8 Department of Obstetrics & Gynaecology, UMC St Radboud, Nijmegen, The Netherlands; 9 Department of Obstetrics & Gynaecology, MUMC, Maastricht, The Netherlands; 10 Department of Obstetrics & Gynaecology, Deventer Ziekenhuis, Deventer, The Netherlands; 11 Department of Obstetrics & Gynaecology, Isala Klinieken, Zwolle, The Netherlands; 12 Department of Obstetrics & Gynaecology, St. Antonius Ziekenhuis, Nieuwegein, The Netherlands; 13 Department of Obstetrics & Gynaecology, Onze Lieve Vrouwe Gasthuis, Amsterdam, The Netherlands; 14 Department of Obstetrics & Gynaecology, Atrium Medical Centre, Heerlen, The Netherlands; 15 Department of Obstetrics & Gynaecology, Catharina Ziekenhuis, Eindhoven, The Netherlands; 16 Department of Obstetrics & Gynaecology, Jeroen Bosch Ziekenhuis, ‘s-Hertogenbosch, The Netherlands

**Keywords:** Heavy menstrual bleeding, Endometrial ablation, LNG-IUS

## Abstract

**Background:**

Heavy menstrual bleeding is an important health problem. Two frequently used therapies are the levonorgestrel intra-uterine system (LNG-IUS) and endometrial ablation. The LNG-IUS can be applied easily by the general practitioner, which saves costs, but has considerable failure rates. As an alternative, endometrial ablation is also very effective, but this treatment has to be performed by a gynaecologist. Due to lack of direct comparison of LNG-IUS with endometrial ablation, there is no evidence based preferred advice for the use of one of these treatment possibilities.

**Method/design:**

A multicenter randomised controlled trial, organised in a network infrastructure in the Netherlands in which general practitioners and gynaecologists collaborate.

Women ≥ 34 years with heavy menstrual bleeding, a Pictorial Blood Assessment Chart (PBAC) score exceeding 150 points and no future child wish can participate in the trial. After informed consent, women will be randomised to a strategy starting with a levonorgestrel releasing intrauterine system or a strategy starting with endometrial ablation.

The primary outcome is the PBAC score at 24 months of follow-up. Secondary outcomes are patient satisfaction, complications, number of re-interventions, menstrual bleeding pattern, quality of life, sexual function, sick leave and costs. As predictors of effect of intervention we also meaure level of coagulation factors.

**Discusson:**

This study, considering both effectiveness and cost effectiveness of LNG-IUS versus endometrial ablation may well improve care for women with heavy menstrual bleeding.

**Trial registration:**

Dutch trial register, number NTR2984

## Background

Heavy menstrual bleeding is a frequent problem that affects many premenopausal women in the Netherlands and is the most important reason for a visit a the outpatient department of gynaecology [1,2]. Each year one in 20 women between 30 to 49 years of age consult their general practitioner (GP) with heavy menstrual bleeding. Hormonal treatment with the oral contraceptive pill or the levonorgestrel releasing intra uterine system (LNG-IUS) or non-hormonal treatment with tranexamic acid or non-steroidal anti-inflammatory drugs (NSAID) are advised as treatments of first choice. Nevertheless 77% of the women are not willing to continue their treatment and often end up undergoing other treatment or even surgery [3,4].

Hysterectomy is a definitive solution for the treatment of heavy menstrual bleeding, and in 2010 11,038 women underwent a hysterectomy due to bleeding disorders in the Netherlands [5]. Recently it was reported that hysterectomy should be considered the preferred strategy for the treatment of heavy menstrual bleeding based on cost-effectiveness [6]. Nevertheless, it is a major surgical procedure and has significant physical complications and social and economic costs [7]. A significant number of women with heavy menstrual bleeding who seek treatment will not benefit from, or will not wish to continue the medical treatment and are keen to preserve their uterus [3,8]. Many women opt for a less invasive treatment, even when they are informed of the fact that success is not always assured [1,9].

Two frequently used minimally invasive treatment options for heavy menstrual bleeding are the LNG-IUS and endometrial ablation.

Intrauterine devices were initially introduced as contraceptives, but after the addition of progestagen (LNG-IUS) these devices also reduce menstrual bleeding effectively. The local release of levonorgestrel in the uterine cavity suppresses endometrial growth. A systematic review on the effectiveness of the LNG-IUS in heavy menstrual bleeding concluded that the reduction of menstrual blood loss was 79-96% in the LNG-IUS group [10-13]. In women with heavy menstrual bleeding who presented to primary care providers, the LNG-IUS was more effective than usual medical treatment in reducing the effect of heavy menstrual bleeding on quality of life [14]. However, up to 60% of women discontinue LNG-IUS within 5 years because of unscheduled bleeding, pain, and/or systemic progestogenic side-effects [15]. In the Royal College of Obstetricians and Gynaecologists (RCOG) guideline on heavy menstrual bleeding, the use of the LNG-IUS is the first therapeutic option when drug treatment has failed. This is not based on proven cost-effectiveness [15].

Endometrial destruction techniques, which aim to destroy or remove the endometrial tissue, have become alternatives to hysterectomies. In 1991 in the Netherlands 21,433 hysterectomies were performed, a number that was reduced to 16,320 in 1998 and just above 11,000 in 2010, which was partly due to the start of using endometrial ablation [16]. Approximately 47-58% percent of women report an amenorrhea after treatment with the bipolar ablation device [5,17-20]. The satisfaction rates of endometrial ablation have been evaluated by several randomised controlled trials and is showing a rate of about 90% [19,21,22]. Ablation techniques result in shorter duration of surgery, shorter hospital stay and quicker recovery time compared to hysterectomy.

Seven trials compared the LNG- IUS with transcervical resection of the endometrium or balloon abaltion. In the meta-analyses a significant lower mean pictorial blood chart score (PBAC) was reported for all women and a significant lower mean reduction in pictorial blood chart score was reported in the surgical group. One study showed a significantly lower median PBAC score in the LNG-IUS group at nine months and one year follow-up 23. Further outcomes showed no difference in satisfaction rates, amenorrhea rates, duration of menstruation, further surgical treatment or quality of life (QoL). Nevertheless, the studies are small, most studies have a short period of follow-up and contain a lot of non-compliance, which makes interpretation of outcomes difficult [12,20,23-27].

Consequently, usual care in the Netherlands implies one of these two strategies for the treatment of heavy menstrual bleeding if drug therapy fails. The strategy LNG-IUS means prescription and placement by the general practitioner (GP) or gynaecologist without anaesthesia. The strategy endometrial ablation implies performing an endometrial ablation by a gynaecologist in day-care or outpatient clinic with or without general anaesthesia. Due to lack of sufficiently powered studies directly comparing LNG-IUS with endometrial ablation, there is currently no evidence based advice for the use of one of these treatment possibilities.

We propose a randomised controlled trial in which these two strategies are compared in the treatment of heavy menstrual bleeding. The study will focus on (cost-) effectiveness, patient satisfaction and QoL.

## Methods/design

### Objective

The aim of this study is to assess the effects and cost-effectiveness of a strategy starting with LNG-IUS versus a strategy starting with endometrial ablation in women with heavy menstrual bleeding. This study will also evaluate QoL, sexual function and as predictors of effect of intervention we also measure level of coagulation factors.

### Trial design

This study is a multicenter randomised controlled trial with an economic evaluation alongside it. It will be performed by the Dutch Consortium for Studies in Women’s Health and the department of General Practice of the University Medical Center Groningen. The study will be organized in a network infrastructure in which GPs and gynaecologists collaborate. Participating hospitals can be district, teaching, third referral or university hospitals. The study is conducted according to the principles of the Declaration of Helsinki and in accordance with the Medical Research Involving Human Subjects Act (WMO) and has been approved by the ethics committee of the Academic Medical Centre Amsterdam (ref. no MEC 10/183). The protocol is registered in the Dutch trial register, number NTR2984.

### Eligibility criteria

Women ≥ 34 years suffering from heavy menstrual bleeding, with a PBAC score exceeding 150 points can participate in the trial.

Women who might want to get pregnant in the future will be excluded as an endometrial ablation is an absolute contraindication for pregnancies. Other exclusion criteria are abnormal cervix cytology in the past 5 years, a sounding length of more than 10 cm, intracavitary fibroids or polyps or large intramural fibroids determined by either a transvaginal ultrasound (TVU) or a bimanual vaginal examination.

### Patient recruitment, randomization and collection of data

Eligible patients are identified by the gynaecologist in the participating hospitals or by participating general practitioners. Before entry into the study, inclusion and exclusion criteria are checked by research nurses and if women are eligible they will also be counseled by these experienced research nurses. They will be informed about the aims, methods, reasonably anticipated benefits and potential hazards of the study. We will ask separate informed consent for taking and analyzing blood on levels of coagulation factors. After given written informed consent women will be randomised to a strategy starting with a LNG-IUS or a strategy starting with endometrial ablation. Randomisation is performed by accessing a web-based randomisation program. We will stratify for inclusion by general practitioner or by gynaecologist. Patients will be randomised into two groups in a ratio of 1:1, using permuted block randomization with a variable block size.

Participants will be given a computer generated numeric code. Data handling will be done anonymously, with the patient code only available to the local investigator and the research nurse working in the local centre. At the local centres, baseline data and follow-up data collection is the responsibility of the specialised research nurse. Data will be collected using a website dedicated to studies in the Dutch Consortium for women’s health and reproductive medicine studies (http://www.studies-obsgyn.nl). In accordance with guidelines of the Dutch Federation of University Medical Centres (NFU) the data will be kept for 20 years.

### Interventions

The LNG-IUS can be placed either by a GP or by a gynaecologist. In the endometrial ablation group, a second generation bipolar ablation technique (Novasure) will be performed by a gynaecologist.

### Measurements and follow-up

Before randomization patients are asked to fill out a PBAC. Prior to start of the treatment, participating patients will fill in two questionnaires measuring health-related QoL: the Short Form-36 (SF-36), a validated GP and the Shaw heavy menstrual bleeding questionnaire, a disease specific questionnaire, which we validated linguistically. Furthermore, patients are asked to complete questionnaires to measure sexual functioning: the Female Sexual Function Index (FSFI), Female Sexual Distress Scale (FSDS) and a questionnaire to measure (indirect) costs. Baseline demographic characteristics, medical history and menstruation pattern are recorded in a Case Record Form (CRF).

Patients will fill in the PBAC and the same questionnaires used at baseline at 3, 6, 12 and 24 months after randomised. Since we compare strategies starting with LNG-IUS or endometrial ablation, patients who are not satisfied with the (randomised) treatment effect can choose another treatment, or opt for hysterectomy. Nevertheless, they will be asked to complete follow-up questionnaires.

### Outcome measures

Primary outcome is the PBAC score at 24 months of follow-up. Secondary outcomes are patient satisfaction, complications, number of re-interventions, menstrual bleeding pattern, including rates of amenorrhea, QoL, sexual function, sick leave and costs and as predictors of effect of intervention we also measure level of coagulation factors.

### Statistical analysis

#### Sample size

The study is designed as a non-inferiority study. Based on previous studies, we estimate the mean PBAC score at 24 months of follow-up to be 50 points in the LNG-IUS group and 40 points in the endometrial ablation group, with a SD of 40 [23-26].

Previous studies in women with heavy menstrual bleeding have shown that a 50 point difference in PBAC –score between treatments represents a clinically meaningful difference in reduction of menstrual bleeding. In our study we use a non-inferiority marge of 25 points. With this marge and estimated mean PBAC-scores of 50 and 40 points in resp. the LNG-IUS and endometrial ablation group, the estimated scores in both treatment groups will be far below the upper range of normal menstrual blood loss. Using a dropout rate of 15%, an alpha error of 2.5% and Beta error of 20%, we need to include 266 patients (133 patients per treatment group).

#### Data analysis

Data will be analysed according to the intention to treat principle. Statistical analysis will be performed using the software Statistical Package for the Social Sciences (SPSS, Inc., Chicago, IL, USA).

Point estimates and 95% confidence intervals will be calculated for different time points.

First, the treatment effect at 24 months of follow-up of a strategy starting with LNG-IUS compared to a strategy starting with endometrial ablation will be investigated. For not normally distributed continuous variables, differences between groups will be tested with the Mann–Whitney U test. Differences between categorical variables will be tested with the Chi-square test or Fisher’s exact test. Secondary, to assess the treatment effect over time, and the interaction of the intervention effect, we will use a mixed effects model. Time to re-intervention will be compared with Kaplan Meier analysis and Cox regression.

The analysis of the influence of lower levels of coagulation on the treatment effect of LNG-IUS and endometrial ablation will be explorative.

### Economic evaluation

The economic analysis will be conducted from a societal perspective including direct medical and direct and indirect non-medical costs. Relevant direct costs components that will be taken into account are costs of the LNG-IUS and endometrial ablation, interventions for complications, hospital admission and home care, consisting of both professional care as well as informal care. Sick leave and loss of productivity at work will be taken into account as indirect non-medical costs.

The economic evaluation will be designed as a cost-effectiveness analysis, with the costs per treatment resulting in a reduction of menstrual blood loss as an outcome measure. We will also perform a cost-utility analysis expressing the incremental costs per Quality Adjusted Life Years based on the SF-36. Robustness of the results (costs and health outcomes) for various assumptions and parameter estimates will be explored in sensitivity analyses and visualized in ICER-graphs and cost-effectiveness acceptability curves.

## Discussion

In view of its high prevalence, an optimal treatment for heavy menstrual bleeding is of utmost importance. Usual care in The Netherlands implies two strategies for the treatment of heavy menstrual bleeding if drug therapy fails: first, there is the LNG-IUS that can be applied easily by the GP, which saves costs, but has considerable failure rates. As an alternative, endometrial ablation is also very effective, but this treatment has to be performed by a gynaecologist. Large randomised controlled trials comparing LNG-IUS with endometrial ablation are lacking and hence no preferred advice for the use of one of these treatment possibilities is available. When we estimate that treatment for heavy menstrual bleeding involves 10,000 women annually, there can be a potential saving from implementing the best strategy as appears from the proposed study. Therefore, a direct comparison of both strategies on cost-effectiveness is essential to determine which strategy should be advocated.

This is the first study comparing LNG-IUS and endometrial ablation which will be organized in a network infrastructure in which both GP’s and gynaecologists collaborate. It has got the largest study population compared to other trials and has a longterm follow-up of 2 years. Besides the objective outcome PBAC we also measure patient satisfaction, QoL and sexual function. Due to this study design and outcome measures, the results will be applicable for a large group of women suffering from heavy menstrual bleeding.

## Competing interests

The authors declare that they have no competing interests.

## Authors’ contributions

MH, MvdB, MBo, PG, JD, MBe, HM, BM were involved in conception and design of the study. MH and MvdB drafted the first manuscript. All other authors are involved in patient recruitment and data collection. All authors edited the manuscript and read and approved the final draft.

## Pre-publication history

The pre-publication history for this paper can be accessed here:

http://www.biomedcentral.com/1472-6874/13/32/prepub

## References

[B1] Palep-Singh M Prentice A Epidemiology of abnormal uterine bleeding Best Pract Res Clin Obstet Gynaecol 2007 21 6 887 890 10.1016/j.bpobgyn.2007.03.012 17936074

[B2] Shapley M Jordan K Croft PR An epidemiological survey of symptoms of menstrual loss in the community Br J Gen Pract 2004 54 502 359 363 15113519 PMC1266170

[B3] Lethaby AE Cooke I Rees M Progesterone or progestogen-releasing intrauterine systems for heavy menstrual bleeding Cochrane Database Syst Rev 2005 4 CD002126 10.1002/14651858.CD002126.pub2 16235297

[B4] Cooper K Lee A Chien P Raja E Timmaraju V Bhattacharya S Outcomes following hysterectomy or endometrial ablation for heavy menstrual bleeding: Retrospective analysis of hospital episode statistics in scotland BJOG 2011 118 10 1171 1179 10.1111/j.1471-0528.2011.03011.x 21624035

[B5] Prismant Dutch Hospital Data 2009

[B6] Roberts TE Tsourapas A Middleton LJ Hysterectomy, endometrial ablation, and levonorgestrel releasing intrauterine system (mirena) for treatment of heavy menstrual bleeding: Cost effectiveness analysis BMJ 2011 342 d2202 10.1136/bmj.d2202 21521730 PMC3082380

[B7] Abbott JA Garry R The surgical management of menorrhagia Hum Reprod Update 2002 8 1 68 78 10.1093/humupd/8.1.68 11866242

[B8] Shaw RW Assessment of medical treatments for menorrhagia Br J Obstet Gynaecol 1994 101 Suppl 11 15 18 8043556 10.1111/j.1471-0528.1994.tb13690.x

[B9] Nagele F Rubinger T Magos A Why do women choose endometrial ablation rather than hysterectomy? Fertil Steril 1998 69 6 1063 1066 10.1016/S0015-0282(98)00082-X 9627293

[B10] Lahteenmaki P Haukkamaa M Puolakka J Open randomised study of use of levonorgestrel releasing intrauterine system as alternative to hysterectomy BMJ 1998 316 7138 1122 1126 10.1136/bmj.316.7138.1122 9552948 PMC28513

[B11] Milsom I Andersson K Andersch B Rybo G A comparison of flurbiprofen, tranexamic acid, and a levonorgestrel-releasing intrauterine contraceptive device in the treatment of idiopathic menorrhagia Am J Obstet Gynecol 1991 164 3 879 883 10.1016/S0002-9378(11)90533-X 1900665

[B12] Crosignani PG Vercellini P Mosconi P Oldani S Cortesi I De Giorgi O Levonorgestrel-releasing intrauterine device versus hysteroscopic endometrial resection in the treatment of dysfunctional uterine bleeding Obstet Gynecol 1997 90 2 257 263 10.1016/S0029-7844(97)00226-3 9241305

[B13] Irvine GA Campbell-Brown MB Lumsden MA Heikkila A Walker JJ Cameron IT Randomised comparative trial of the levonorgestrel intrauterine system and norethisterone for treatment of idiopathic menorrhagia Br J Obstet Gynaecol 1998 105 6 592 598 10.1111/j.1471-0528.1998.tb10172.x 9647148

[B14] Gupta J Kai J Middleton L Levonorgestrel intrauterine system versus medical therapy for menorrhagia N Engl J Med 2013 368 2 128 137 10.1056/NEJMoa1204724 23301731

[B15] Ewies AA Levonorgestrel-releasing intrauterine system–the discontinuing story Gynecol Endocrinol 2009 25 10 668 673 10.1080/09513590903159656 19657812

[B16] Middleton LJ Champaneria R Daniels JP Hysterectomy, endometrial destruction, and levonorgestrel releasing intrauterine system (mirena) for heavy menstrual bleeding: Systematic review and meta-analysis of data from individual patients BMJ 2010 341 c3929 10.1136/bmj.c3929 20713583 PMC2922496

[B17] Gallinat A Nugent W NovaSure impedance-controlled system for endometrial ablation J Am Assoc Gynecol Laparosc 2002 9 3 283 289 10.1016/S1074-3804(05)60405-7 12101323

[B18] Clark TJ Samuels N Malick S Middleton L Daniels J Gupta J Bipolar radiofrequency compared with thermal balloon endometrial ablation in the office: a randomised controlled trial Obstet Gynecol 2011 117 5 1228 21705927 10.1097/AOG.0b013e3182175ba5

[B19] Bongers MY Bourdrez P Mol BW Heintz AP Brolmann HA Randomised controlled trial of bipolar radio-frequency endometrial ablation and balloon endometrial ablation BJOG 2004 111 10 1095 1102 10.1111/j.1471-0528.2004.00253.x 15383112

[B20] Marjoribanks J Lethaby A Farquhar C Surgery versus medical therapy for heavy menstrual bleeding Cochrane Database Syst Rev 2006 2 CD003855 10.1002/14651858.CD003855.pub2 16625593

[B21] Penninx JP Herman MC Mol BW Bongers MY Five-year follow-up after comparing bipolar endometrial ablation with hydrothermablation for menorrhagia Obstet Gynecol 2011 118 6 1287 1292 10.1097/AOG.0b013e318236f7ed 22105257

[B22] Bongers MY Mol BW Thermal balloon ablation versus endometrial resection for treatment of abnormal uterine bleeding Hum Reprod 2000 15 6 1424 1425 10.1093/humrep/15.6.1424 10831585

[B23] Shaw RW Symonds IM Tamizian O Chaplain J Mukhopadhyay S Randomised comparative trial of thermal balloon ablation and levonorgestrel intrauterine system in patients with idiopathic menorrhagia Aust N Z J Obstet Gynaecol 2007 47 4 335 340 10.1111/j.1479-828X.2007.00747.x 17627692

[B24] Istre O Trolle B Treatment of menorrhagia with the levonorgestrel intrauterine system versus endometrial resection Fertil Steril 2001 76 2 304 309 10.1016/S0015-0282(01)01909-4 11476777

[B25] Barrington JW Arunkalaivanan AS Abdel-Fattah M Comparison between the levonorgestrel intrauterine system (LNG-IUS) and thermal balloon ablation in the treatment of menorrhagia Eur J Obstet Gynecol Reprod Biol 2003 108 1 72 74 10.1016/S0301-2115(02)00408-6 12694974

[B26] Busfield RA Farquhar CM Sowter MC A randomised trial comparing the levonorgestrel intrauterine system and thermal balloon ablation for heavy menstrual bleeding BJOG 2006 113 3 257 263 10.1111/j.1471-0528.2006.00863.x 16487195

[B27] Soysal M Soysal S Ozer S A randomised controlled trial of levonorgestrel releasing IUD and thermal balloon ablation in the treatment of menorrhagia Zentralbl Gynakol 2002 124 4 213 219 10.1055/s-2002-32434 12080483

